# Translating color fundus photography to indocyanine green angiography using deep-learning for age-related macular degeneration screening

**DOI:** 10.1038/s41746-024-01018-7

**Published:** 2024-02-12

**Authors:** Ruoyu Chen, Weiyi Zhang, Fan Song, Honghua Yu, Dan Cao, Yingfeng Zheng, Mingguang He, Danli Shi

**Affiliations:** 1 Experimental Ophthalmology, School of Optometry, The Hong Kong Polytechnic University, Kowloon Hong Kong SAR, China; 2 Research Centre for SHARP Vision, The Hong Kong Polytechnic University, Kowloon Hong Kong SAR, China; 3 Department of Ophthalmology, Guangdong Academy of Medical Sciences, Guangdong Provincial People’s Hospital, Southern Medical University, Guangzhou, China; 4 State Key Laboratory of Ophthalmology, Zhongshan Ophthalmic Center, Sun Yat-sen University, Guangdong Provincial Key Laboratory of Ophthalmology and Visual Science, Guangdong Provincial Clinical Research Center for Ocular Diseases, Guangzhou, China; 5 Centre for Eye and Vision Research (CEVR), 17W Hong Kong Science Park, Hong Kong SAR, China

**Keywords:** Macular degeneration, Medical imaging

## Abstract

Age-related macular degeneration (AMD) is the leading cause of central vision impairment among the elderly. Effective and accurate AMD screening tools are urgently needed. Indocyanine green angiography (ICGA) is a well-established technique for detecting chorioretinal diseases, but its invasive nature and potential risks impede its routine clinical application. Here, we innovatively developed a deep-learning model capable of generating realistic ICGA images from color fundus photography (CF) using generative adversarial networks (GANs) and evaluated its performance in AMD classification. The model was developed with 99,002 CF-ICGA pairs from a tertiary center. The quality of the generated ICGA images underwent objective evaluation using mean absolute error (MAE), peak signal-to-noise ratio (PSNR), structural similarity measures (SSIM), etc., and subjective evaluation by two experienced ophthalmologists. The model generated realistic early, mid and late-phase ICGA images, with SSIM spanned from 0.57 to 0.65. The subjective quality scores ranged from 1.46 to 2.74 on the five-point scale (1 refers to the real ICGA image quality, Kappa 0.79–0.84). Moreover, we assessed the application of translated ICGA images in AMD screening on an external dataset (*n* = 13887) by calculating area under the ROC curve (AUC) in classifying AMD. Combining generated ICGA with real CF images improved the accuracy of AMD classification with AUC increased from 0.93 to 0.97 (*P* < 0.001). These results suggested that CF-to-ICGA translation can serve as a cross-modal data augmentation method to address the data hunger often encountered in deep-learning research, and as a promising add-on for population-based AMD screening. Real-world validation is warranted before clinical usage.

## Introduction

Age-related macular degeneration (AMD) is the leading cause of central vision loss in the aging population^1^, mainly consisting of atrophic (“dry”) AMD and neovascular (“wet”) AMD. Dry AMD may progress to wet AMD, which is characterized by central choroidal neovascularization (CNV), resulting in hemorrhaging within the macular region and profound visual impairment. Effective and accurate AMD screening tools are urgently needed, especially as the aging population intensifies^2^. Over the past few years, applying color fundus (CF) photography to develop deep learning algorithms for automated AMD screening is of great interest^3–5^. However, it is worth mentioning that only CF images provide limited information in the real clinical scenario, because of the unstable image quality and common characteristics shared by several chorioretinal diseases on CF images.

Indocyanine green angiography (ICGA) is a well-established fundus imaging technique for detecting and distinguishing AMD from other chorioretinal diseases^6–9^. Compared to CF images, ICGA owns its unique advantages in dynamically visualizing deeper choroidal vasculature and lesions behind retinal pigment epithelium^10,11^. However, ICGA is an invasive imaging modality with potential adverse reactions, including nausea, vomiting, hypotensive shock, etc^12–15^. In addition, the complex operating procedures impede its widespread implementation in clinical settings. Limited and imbalanced ICGA images pose challenges to the development of relevant automated AMD detection models.

Recently, generative adversarial networks (GANs) have showcased remarkable performance in image-to-image translation through two competing types of deep neural networks^16–18^, which inspire generating multiple ICGA images from non-invasive CF images. Several exploratory studies have verified the feasibility of cross-modality image translation in the ophthalmic field via GANs, such as CF to fundus fluorescein angiography (FFA) translation^19,20^. However, few studies have considered detailed phase features and diverse pathological lesions during the process of generating angiographic images, which was essential in diagnosing chorioretinal vasculopathy. Besides, there is no study aimed at achieving CF-to-ICGA translation.

The purpose of our study is to train a GAN-based model for generating realistic ICGA images from CF images using a large clinical dataset and to validate its robustness in AMD screening via external dataset. Our CF-to-ICGA algorithm is expected to provide an effective alternative for addressing the inadequacy and imbalance of ICGA images in deep-learning research, while also promoting the advancement of more accurate AMD screening models.

## Results

**Fig. 1:**
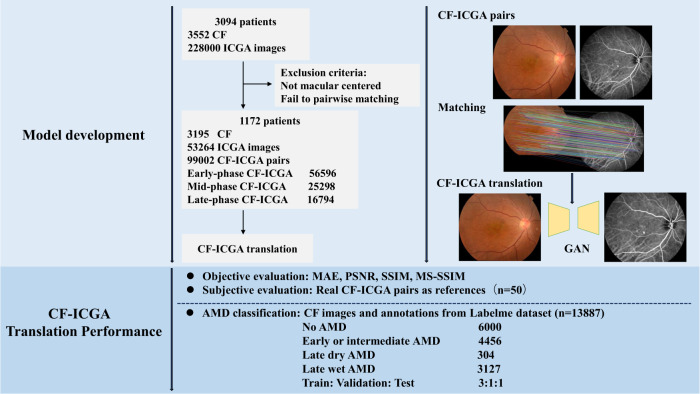
Flow chart of the study. GAN generative adversarial networks, CF color fundus photography, ICGA indocyanine green angiography, AMD age-related macular degeneration, MAE mean absolute error, PSNR peak signal-to-noise ratio, SSIM structural similarity measures, MS-SSIM multi-scale structural similarity measures.

Images that were not centered on the macula were excluded. The final dataset contains 3195 CF images and 53,264 ICGA images from 1172 patients. Thus, an average of 3 CF images and 45 ICGA images were obtained from each patient. To fully utilize the large number size of ICGA images, we matched each ICGA image with each CF pairwisely (paired images were all from the same patient and visit). After excluding images that failed to be pairwisely matched, we finally yielded 99,002 CF-ICGA pairs for model development, in which there were 56596 pairs in early-phase, 25,298 pairs in mid-phase, and 16,794 in late-phase (Fig. 1). The median (interquartile range) age of the participants was 53.04 (±17.31) years, and 676 (57.7%) were male. The majority of these participates were diagnosed with chorioretinal diseases, including AMD, choroidal neovascularization (CNV), PCV, and pathologic myopia, etc. In the AMD dataset, CF images were classified as no AMD, early or intermediate dry AMD, late dry AMD, and late wet AMD. The study flow chart is shown in Fig. 1. Detailed characteristics of the dataset are presented in Table 1.

**Table 1:** Dataset characteristics

			*N*	No					
**Model development**				normal	AMD	PCV	CNV	PM	Other
Patients		1172		267 (22.8%)	320 (27.3%)	158 (13.5%)	141 (12.0%)	104 (8.9%)	182 (15.5%)
**External validation**									
CF-ICGA pairs		50		Dry AMD	Wet AMD	PCV	CSC	Others	
				12	15	5	6	12	
				(24.0%)	(30.0%)	(10.0%)	(12.0%)	(24.0%)	
**AMD Classification (AMD Dataset)**				No AMD	Early or intermediate dry AMD	Late dry AMD	Late wet AMD		
Train	8331			3600 (43.2%)	2673 (32.1%)	182 (2.2%)	1876 (22.5%)		
Validation	2781			1200 (43.2%)	891 (32.0%)	61 (2.2%)	629 (22.6%)		
Test	2775			1200 (43.2%)	892 (32.1%)	61 (2.2%)	622 (22.4%)		
Total	13887			6000 (43.2%)	4456 (32.1%)	304 (2.2%)	3127 (22.5%)		

*CF* color fundus photography, *ICGA* indocyanine green angiography, *AMD* age-related macular degeneration, *PCV* polypoidal choroidal vasculopathy, *CNV* choroidal neovascularization, *CSC* central serous chorioretinopathy, *N* number of patients/images.

### Objective evaluation

Pixel-wise comparison between the real and CF-translated ICGA was conducted on the internal test set. For the generated early-phase ICGA, the mean absolute error (MAE), peak signal to noise ratio (PSNR), structural similarity measures (SSIM), and multi-scale structural similarity measures (MS-SSIM) were 86.81, 20.01, 0.57, and 0.68. For the generated mid-phase ICGA, the MAE, PSNR, SSIM, and MS-SSIM were 116.94, 21.74, 0.65, and 0.70. For the generated late-phase ICGA, the MAE, PSNR, SSIM, and MS-SSIM were 118.19, 22.83, 0.57, and 0.74. These results are shown in Table 2.

**Table 2:** Objective evaluation of real and translated indocyanine green angiography (ICGA) images

	MAE	PSNR	SSIM	MS-SSIM
Early-phase ICGA	86.81	20.01	0.57	0.68
Mid-phase ICGA	116.94	21.74	0.65	0.70
Late-phase ICGA	118.19	22.83	0.57	0.74

*MAE* mean absolute error, *PSNR* peak signal-to-noise ratio, *SSIM* structural similarity measures, *MS-SSIM* multi-scale structural similarity measures.

### Subjective evaluation

**Fig. 2:**
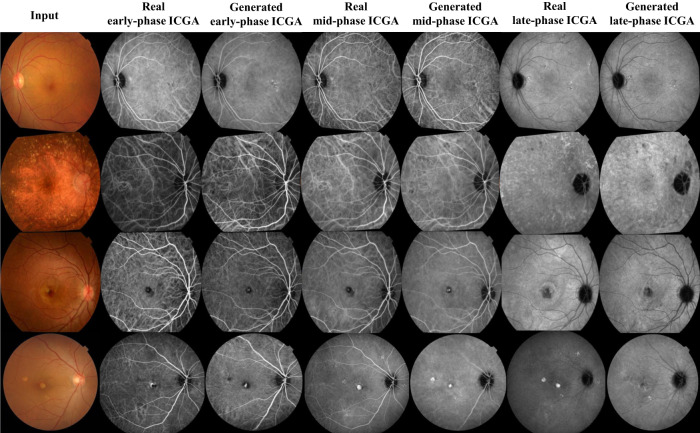
Examples of real and translated indocyanine green angiography (ICGA). 1st row, early dry age-related macular degeneration (AMD), 2nd row, intermediate dry AMD, 3rd row, wet AMD, 4th row, wet AMD. 1 – 3 rows: internal test set, CF were registered with ICGA, rotation occurs during this process. 4th row: external test set.

Example-generated images in the internal and external test sets are shown in Fig. 2. The model efficiently and anatomically achieved CF-ICGA pairwise matching via retinal vascular features, generating realistic ICGA images with detailed structures and lesions. Notably, background noise sometimes present in the real ICGA was learned as unrelated and was effectively excluded in the translated images. The synthesized output images are visually very close to real ones. For the internal test set, a blinded evaluation was conducted using 30 ICGA images with 50% real ICGA images by removing the tags of “Original” and “Generated” on these images. Two experienced ophthalmologists (R.C. and F.S.) selected ICGA images in random order for identification. Among these unlabeled ICGA images, 6.6% and 13.3% generated ICGA images could be differentiated from the real ones. The distinguishing features encompassed the blurry boundary of lesions, strange lesions that are apparently against established clinical knowledge, vascular discontinuity, and the blurry texture of choroidal vessels (Supplementary Fig. 5). For the external test set, the dissimilarities in image characteristics between the training dataset and the external dataset were readily apparent. Consequently, the real and generated ICGA images could be discerned based on image style alone, and nearly all the generated ICGA images could be distinguished from the authentic ones (as illustrated in Fig. 2, 4th row). Thus, we did not conduct blind evaluations in this case.

Image quality assessment, which considers chorioretinal structure and lesions, was based on a five-point scale. The mean (standard deviation [SD]) of the scores for early-phase ICGA was 1.46 (0.76), 2.08 (0.85) for the internal and external test set respectively, assessed by the first grader, and 1.48 (0.76), 2.12 (0.77) by the second grader. The mean (SD) of the scores for mid-phase ICGA were 2.02 (0.84) and 2.56 (0.91) in the internal and external test sets respectively, assessed by the first grader, and 1.94 (0.89), 2.66 (0.98) by the second grader. The mean (SD) of the scores for late-phase ICGA were 2.04 (0.81) and 2.74 (0.82) in the internal and external test sets respectively, assessed by the first grader, and 1.96 (0.78), 2.64 (0.74) by the second grader. Cohen’s kappa values indicate an excellent agreement between the two graders for assessing image quality, with the Kappa value of 0.79, 0.81 for early-phase ICGA, 0.81, 0.80 for mid-phase ICGA, and 0.81, 0.84 for late-phase ICGA in the internal and external test sets, respectively (Table 3). This reflects the high quality of synthesized images for anatomical features (vessel, optic disc, and macula) and lesions (drusen, choroidal neovascular, atrophy, subretinal fluid, and hemorrhage).

**Table 3:** Subjective evaluation of real and translated indocyanine green angiography (ICGA) image quality

	Internal test set (*N* = 50)			External test set (*N* = 50)		
	Rater 1 Mean (SD)	Rater 2 Mean (SD)	Kappa	Rater 1 Mean (SD)	Rater 2 Mean (SD)	Kappa
early-phase ICGA	1.46 (0.76)	1.48 (0.76)	0.79	2.08 (0.85)	2.12 (0.77)	0.81
mid-phase ICGA	2.02 (0.84)	1.94 (0.89)	0.81	2.56 (0.91)	2.66 (0.98)	0.80
late-phase ICGA	2.04 (0.81)	1.96 (0.78)	0.81	2.74 (0.82)	2.64 (0.74)	0.84

*SD* standard deviation.

In the internal and external test sets, 5.3% and 12.0% of the generated ICGA images were of poor quality (>=4 points) due to the following reasons: blurry CF images impact the quality of synthetic ICGA. Besides, choroidal lesions could be sheltered by extremely thick subretinal hemorrhage and scar, resulting in the generation of false negative or positive lesions, as demonstrated in Supplementary Fig. 3.

While this study primarily focused on AMD, Supplementary Fig. 4 provides generation examples for normal fundus and other diseases, such as polypoidal choroidal vasculopathy, central serous chorioretinopathy, pathologic myopia, and punctate inner choroidopathy to further demonstrate the comprehensiveness of this model.

### AMD classification

**Fig. 3:**
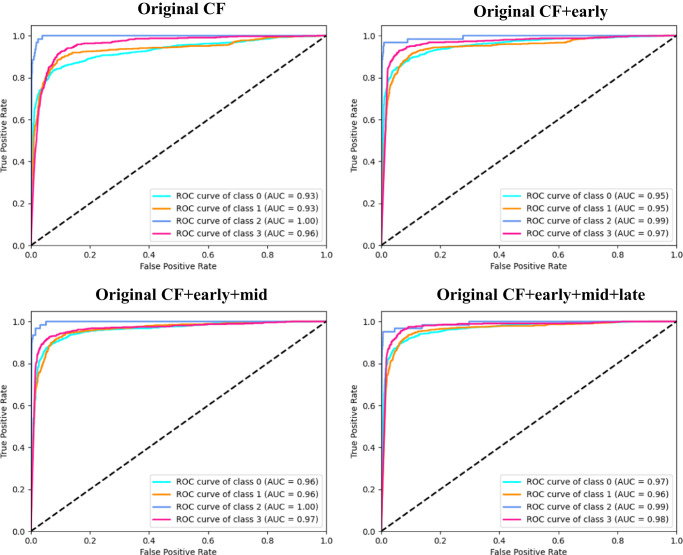
ROC curves of age-related macular degeneration (AMD) classification on the AMD dataset (*n* = 13887) with and without the addition of translated indocyanine green angiography (ICGA) on top of color fundus photography (CF). The classification is based on four categories: 0 = no AMD, 1 = early or intermediate dry AMD, 2 = late dry AMD, and 3 = late wet AMD. 1st row (left) = Original CF, 1st row (right) = Original CF plus generated early-phase ICGA images, 2nd row (left) = Original CF plus generated early-phase plus mid-phase ICGA images, 2nd row (right) = Original CF plus generated early-phase plus mid-phase plus late-phase ICGA images.

**Fig. 4:**
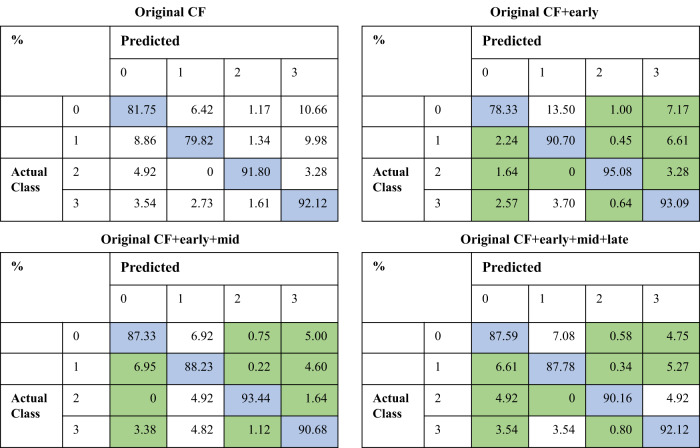
Confusion matrix of age-related macular degeneration (AMD) classification results on the AMD dataset (*n* = 13887) with and without the addition of translated indocyanine green angiography (ICGA) on top of color fundus photography (CF). The classification is based on four categories: 0 = no AMD, 1 = early or intermediate dry AMD, 2 = late dry AMD, and 3 = late wet AMD. 1st row (left) = Original CF, 1st row (right) = Original CF plus generated early-phase ICGA images, 2nd row (left) = Original CF plus generated early-phase plus mid-phase ICGA images, 2nd row (right) = Original CF plus generated early-phase plus mid-phase plus late-phase ICGA images.

The addition of generated ICGA on top of CF significantly improved the accuracy of AMD classification on the inhouse AMD datasets^21^, as illustrated in Table 4 and Fig. 3. The integration of generated ICGA images significantly reduced error rates for AMD categories (Fig. 4). In the test set, 219 (18.3%) non-AMD cases, 180 (20.2%) early or intermediate dry AMD cases, 5 (8.2%) late dry AMD cases, and 49 (7.9%) late wet AMD cases were falsely predicted based on CF alone. Among these false prediction cases, 21.0%-48.9% with no AMD, 52.2–61.1% with early or intermediate dry AMD, 20.0–60.0% with late dry AMD, and 42.9–44.9% with late wet AMD could be accurately predicted after adding generated ICGA images (Table 5 and Supplementary Fig. 6). In addition, significant differences in AMD classification accuracies were found between CF alone and CF+generated early-phase ICGA+generated mid-phase ICGA, as well as CF alone and CF+generated early-phase ICGA+generated mid-phase+generated late-phase ICGA (*P* < 0.001). However, there were no statistical differences between CF alone and CF+early-phase ICGA (*P* = 0.44) (Table 4). In general, these results demonstrated that incorporating synthetic ICGA images with CF significantly enhances AMD classification accuracy.

**Table 4:** Age-related macular degeneration (AMD) classification based on color fundus photography (CF) and CF+ translated indocyanine green angiography (ICGA) images on the AMD dataset (*n* = 13887)

	F1-score	Sensitivity	Specificity	Accuracy	AUC	*P* value*
CF	0.8386	0.8368	0.9323	0.8368	0.9312	
CF+early	0.8601	0.8598	0.9428	0.8598	0.9407	0.4400
CF+early+mid	0.8854	0.8850	0.9466	0.8850	0.9632	<0.0001**
CF+early+mid+late	0.8875	0.8872	0.9474	0.8872	0.9688	<0.0001**

*The distinctions of ROC curves were evaluated using the bootstrap method in the R package pROC between the following scenarios: CF alone versus CF+generated early-phase ICGA images, CF alone versus CF+generated early-phase ICGA+generated mid-phase ICGA, CF alone versus generated early-phase ICGA+generated mid-phase ICGA+generated late-phase ICGA. **P < 0.05. The AMD dataset, train: validation: test = 6:2:2.

**Table 5:** Comparison of false prediction cases on different age-related macular degeneration (AMD) categories based on color fundus photography (CF) only but were correctly predicted after the addition of translated indocyanine green angiography (ICGA) images in the test set of AMD dataset (*n* = 2775)

	No AMD	Early or intermediate dry AMD	Late dry AMD	Late wet AMD
Total, *N*	1200	892	61	622
False prediction, *N* (%)				
CF only	219 (18.3)	180 (20.2)	5 (8.2)	49 (7.9)
Correct prediction rate after adding generated ICGA images, *N* (%)				
CF+early	46 (21.0)	110 (61.1)	2 (40.0)	22 (44.9)
CF+early+mid	107 (48.9)	94 (52.2)	1 (20.0)	21 (42.9)
CF+early+mid+late	101 (46.1)	94 (52.2)	3 (60.0)	21 (42.9)

*N* number of cases, *CF+early* CF+generated early-phase ICGA images, *CF+mid* CF+generated mid-phase ICGA images, *CF+late* CF+generated late-phase ICGA images.

## Discussion

In the present study, we innovatively demonstrated that high-resolution ICGA images could be synthesized based on CF images using GANs. The reliability of this model in generating authentic ICGA images has been proven through both internal and external validation. Additionally, we have illustrated that the integration of the translated ICGA images with CF images significantly improves the accuracy of AMD screening. Our study not only established the feasibility of predicting choroidal abnormalities more accurately from the more accessible CF images via GANs but also introduced a cross-modality approach to augment data for AMD-related deep learning research.

Image-to-image translation has garnered significant attention within the domain of fundus multimodal imaging systems^16,22,23^. The generation of realistic ICGA images from CF images posed a significant challenge due to the masking effect of the retinal pigment epithelium and the intricate anatomical structure of the choroid. To address this challenge, our algorithm leveraged the power of Pix2pix HD, which belongs to the conditional GANs family, and demonstrates excellent performance in image-to-image translation via reducing adversarial loss and pixel-reconstruction error^24^. Its strong ability in denoising, super-resolution, and feature extraction has provided a robust foundation for accurate CF-to-ICGA translation^25^. Considering exudation, hemorrhage, and other abnormalities may result in poor generation, we applied an additional Gradient Variance Loss to generate high-resolution details with sharp edges by minimizing the distance between the computed variance maps and enforcing the model to produce high-variance gradient maps^26^.

ICGA is a unique modality for detecting choroidal abnormalities because of its strong penetration and high contrast characteristics, offering much more information on choroidal circulation than other non-invasive approaches, such as CF and optic coherence tomography angiography (OCTA)^27–29^. In the current study, the detailed phase information of ICGA images, such as cut-off time and fluorescence characteristics of each phase were considered in model training. The current GAN-based model could authentically generate ICGA images of early, medium, and late phases respectively from a single CF image, suggesting an effective alternative for observing choroidal lesions dynamically and non-invasively. Our results illustrated that the addition of generated ICGA images significantly improves the accuracy of AMD classification within an external dataset compared to CF alone. The comparable performance between CF and CF plus early-phase ICGA may result from the limited information provided by early-phase ICGA alone.

AMD shares many common features with PCV, but significant disparities in treatment response and prognosis are also found in these two diseases, emphasizing the need for distinguishing PCV from AMD^30,31^. In our study, the branch vascular networks and polyps, which are the landmarks for diagnosing PCV, can be realistically generated (Supplementary Fig. 4, 1st row). In addition, normal fundus and characteristic lesions of other chorioretinal diseases could also be well translated on ICGA images (Supplementary Fig. 4, 2rd–5th row). Our findings also demonstrated that 42.9% to 60.0% of the cases misclassified by CF alone could be correctly predicted after adding all-phase generated ICGA, further showcasing that these synthesized ICGA can effectively reduce classification errors and facilitate more accurate AMD screening in the current research background. Nevertheless, generative technology is still at the exploratory stage in ophthalmic research with yet unclear clinical relevance^16,32^. Prospective trials and clinician-engineer collaboration are necessary to prove whether the application of synthetic images will optimize clinical practice.

The inadequacy of ICGA images represents a challenge in deep learning research aimed at developing automated tools for detecting chorioretinal conditions. In the past few decades, GANs-based technology has been introduced to expand datasets and protect patient privacy via generating realistic images, especially of uncommon diseases^33–35^. Several studies have reported that integrating synthetic images could enhance the performance of machine-learning models in the ophthalmic field^36–38^. In addition, synthetic data could be incorporated into various research tasks, such as lesion segmentation, image denoising, super-resolution, etc^16^. Thus, translated ICGA images may also expected to address data shortages and imbalances, enabling low-shot or zero-shot training in ICGA relevant deep-learning model. The cross-modality image translation may be a good choice in maximizing the potential of existing datasets for the development of deep learning systems.

This research also has limitations. Firstly, the presence of extremely thick subretinal hemorrhage and scars, along with blurry CF images can potentially impact the quality of the generated ICGA images, resulting in false negative or false positive lesions due to incorrect matching of blurry images in translation procedure. Secondly, though a large-scale dataset was utilized for training in the current study, more variable real-world datasets are critical for improving the diversity and applicability of generated ICGA images. Most importantly, the current research aims to explore the authenticity and limitations of synthetic images in a controlled research context, future validation is warranted to evaluate whether synthetic ICGA images could assist clinical decision-making.

In conclusion, we innovatively developed a deep-learning model for generating ICGA images of early, medium, and late phases from CF images. The algorithm showed high authenticity in generating anatomical structures and pathological lesions in both internal and external datasets. These findings highlight the potential of the CF-to-ICGA model as a valuable approach for evaluating chorioretinal circulation and abnormalities non-invasively, as well as a promising tool for overcoming data shortages in machine-learning model training. Further clinical trials are required to translate this research discovery into clinical benefit in real-world practice.

## Methods

### Data

We collected a total of 3552 CF images and 228,000 ICGA images from a tertiary center between 2016 and 2019. These images were sourced from 3094 patients who had been diagnosed with a range of ocular diseases, including age-related macular degeneration (AMD), polypoidal choroidal vasculopathy (PCV), choroidal neovascularization (CNV), central serous chorioretinopathy (CSCR), pathologic myopia (PM), and ocular inflammatory diseases, etc. All patient data underwent anonymization and de-identification processes. The CF images were captured using Topcon TRC-50XF and Zeiss FF450 Plus (Carl Zeiss, Inc., Jena, Germany) cameras, with resolutions spanning from 1110 × 1467 to 2600 × 3200. The ICGA images were acquired using Heidelberg Spectralis cameras (Heidelberg, Germany) at a resolution of 768 × 768.

To assess the reliability of our model, we conducted a retrospective collection of 50 paired CF and ICGA images specifically focusing on choroidal conditions from Guangdong Provincial People’s Hospital. The CF images were captured using Topcon TRC-50XF cameras, while the ICGA images were obtained using Heidelberg Spectralis cameras from Heidelberg, Germany.

To assess the efficacy of translated ICGA images in enhancing AMD detection, we utilized a separated dataset procured from a web-based, cloud-sourcing platform situated in Guangzhou, China^21^. This dataset encompassed macular and disc-centered CF images sourced from 36 ophthalmology departments, optometry clinics, and screening facilities spanning various regions of China. The grading of AMD followed the Beckman clinical classification system which involved categorizing patients into groups based on the absence of AMD, early-stage AMD, intermediate-stage AMD, and late-stage AMD (atrophic or neovascular), considering the severity and pathological progression^39^. A detailed overview of the dataset characteristics is presented in Table 1.

The study adheres to the tenets of the Declaration of Helsinki. The Institutional Review Board approved the study and individual consent for this retrospective analysis was waived (Approval Number: 2021KYPJ164 – 3).

### CF and ICGA matching

We conducted a pairwise matching process between CF and ICGA images obtained from the same eye and visit. CF and ICGA images captured from the same eye and eye position share common retinal vessel features, which are stable and easily detectable in both modalities. Thus, we utilized the retinal vessel features to achieve CF-ICGA pairwise matching. We applied the Retina-based Microvascular Health Assessment System (RMHAS) segmentation module to extract retinal vessels from CF and ICGA images^40^. The cross-modality matching process follows previous work^41^. Initially, key points from both modalities were identified from the corresponding vessel maps using the AKAZE key point detector through feature matching^42^. Then the RANSAC (random sample consensus) algorithm was utilized to generate homography matrices and eliminate outliers to facilitate registration^43^. In addition, we implemented a validity restriction to ensure the accuracy of the registration. This restriction enforced a rotation scale value between 0.8 and 1.3, and an absolute rotation radian value less than 2 before the warping transformation. Additionally, we filtered out image pairs with poor registration performance by setting a dice coefficient threshold of 0.5, which was determined empirically based on our dataset during experiment.

### CF to ICGA translation

In our training process, we utilized CF images as input and the corresponding early-phase, mid-phase, and late-phase ICGA images as the ground truth to train three separate models with CF-ICGA pairs. To reduce variation, we established specific time ranges for each phase of ICGA images: 25 s to 3 min for early-phase, 3 to 15 min for mid-phase, and after 15 min for late-phase. The dataset was split into three sets: training, validation, and testing, following a 6:2:2 ratio based on patient level. During training, the images were resized to 512 × 512 and fed into pix2pixHD^44^, a popular GAN model consisting of generator G, which synthesizes candidate samples based on the data distribution of the original dataset, and a discriminator D, which distinguishes the synthesized candidate samples from the original samples. The discriminator employed a multi-scale convolutional neural network that divided the image into patches and evaluated the fidelity of each patch. This approach contributed to the generation of high-resolution ICGA images that closely resembled the real ones. To enhance the generation of high-frequency components, such as chorioretinal structure and lesions, we incorporated Gradient Variance Loss to prevent overfitting^26^. Additionally, extensive data augmentations were applied during training, including random resized crops within a scale range of 0.3–3.5, random horizontal or vertical flipping, and random rotations within a range of 0–45 degrees. Individual CF images and their corresponding generated ICGA images all underwent the same augmentation process. The models were trained with a batch size of 4 and a learning rate of 0.0002. Each training session was preset to run for a total of 50 epochs, ensuring an adequate number of iterations for convergence and optimization in our experiments.

Assessment of CF-to-ICGA Translation Performance.

### Objective evaluation

For the evaluation of image authenticity, we employed four standard objective measures widely used in image generation for our internal test set. MAE computes the average absolute pixel difference between the generated image and the corresponding real image. It quantifies the overall discrepancy in pixel values, indicating the level of fidelity in generating accurate details. PSNR is the approximation of human perception regarding reconstruction quality. It measures the ratio between the maximum possible power of a signal and the power of the noise interfering with it. SSIM^45^ assesses the structural similarity between images, with a value of 1 representing complete similarity and 0 indicating no similarity. SSIM provides insights into the visual resemblance and coherence between the generated and real images. MS-SSIM^46^ supplies more flexibility in incorporating the variations of viewing conditions and image resolution. The higher the SSIM, MS-SSIM, and PSNR, the better the quality of the generated images.

### Subjective evaluation

Fifty images from the internal and external test sets were randomly assigned to two experienced ophthalmologists (R.C. and F.S.) for visual quality assessment. The ophthalmologists assessed the translated images subjectively, considering factors such as the global similarity, the fidelity of anatomical structures, and the depiction of fluorescence-based pathological lesions, on a scale of 1 to 5 (1 = excellent, 2 = good, 3 = normal, 4 = poor, and 5 = very poor), with score 1 referring to the image quality of the real ICGA image. The detailed grading criteria and examples of different quality were demonstrated in Supplementary Figs. 1 and 2. To determine the agreement between the ophthalmologists, we calculated Cohen’s linearly weighted kappa score^47^. This score ranges from −1 to 1, with values between 0.40 and 0.60 indicating moderate agreement, 0.60 and 0.80 indicating substantial agreement, and 0.80 and 1.00 indicating almost perfect agreement. The inter-rater agreement was assessed based on this evaluation metric. Besides, blind assessment was conducted using ICGA images without tags of “Original” and “Generated” to investigate whether it is possible to reliably distinguish between real and generated ICGA images for ophthalmologists.

### AMD classification

CF-ICGA translation performance was also evaluated using an external AMD dataset, which consists of real CF images and AMD annotations. Our GAN-based model generated corresponding early-phase, mid-phase, and late-phase ICGA images respectively from each real CF image. We did experiments using the Swin-transformer to explore whether the addition of ICGA images generated by our model could improve the classification accuracy of AMD^48^. The same hyperparameters were set in each experiment to classify AMD based on CF, CF+generated early-phase ICGA, CF+generated early-phase+mid-phase ICGA images, and CF+generated early-phase+mid-phase+late-phase ICGA images. Four relevant embedding models were initialized with pretrained weights from ImageNet^49^, and the classifier shared the same training data with the embedding model. Specifically, the features from different images were extracted by Swin-transformer into 512-dimensional embeddings, these embeddings were then concatenated and passed through a fully-connected layer, followed by a softmax layer to obtain the classification output. The dataset was divided into training, validation, and test sets in a ratio of 6:2:2. During training, the images were resized to 512 × 512 and augmented with random horizontal flips and rotations ranging from −30 to 30 degrees. The Adam optimizer with a learning rate of 1e-5 and a batch size of 4 was employed. Each experiment was trained for 30 epochs, and the models with the highest area under the ROC curve (AUC) value on the validation set were selected for testing. The performance of AMD classification was assessed on the test set using various metrics, including F1-score, Sensitivity, Specificity, Accuracy, and AUC. Confusion matrices were utilized to analyze the fine-grained class-level performance as well. Additionally, to verify whether the addition of generated ICGA images could improve AMD prediction performance, we conducted a comparative analysis of ROC curves using the bootstrap method in the R package pROC^50^. Specifically, we examined the distinctions of ROC curves between the following scenarios: CF alone versus CF+generated early-phase ICGA images, CF alone versus CF+generated early-phase ICGA+generated mid-phase ICGA, CF alone versus generated early-phase ICGA+generated mid-phase ICGA+generated late-phase ICGA. Reported *P* values are two-sided and the results were considered statistically significant with *P* < 0.05.

### Supplementary information

Supplementary Fig. 1 – 6

## Data Availability

The data used for model development of this study are not openly available due to reasons of privacy and are available from the corresponding author upon reasonable request. The AMD dataset used for external validation is located on a controlled access data platform. Interested researchers can contact M.H. (mingguang.he@polyu.edu.hk) for more information.

## Code availability

The deep-learning model was developed using PyTorch (http://pytorch.org). We trained the model on an NVIDIA GeForce RTX 3090 card. The code for deep learning model development can be accessed at https://github.com/NVIDIA/pix2pixHD).
