# Supplementary material for: Translating color fundus photography to indocyanine green angiography using deep-learning for age-related macular degeneration screening
Source: NPJ Digit Med. 2024 Feb 12;7:34. doi: 10.1038/s41746-024-01018-7 (PMC10861476; doi:10.1038/s41746-024-01018-7)
Supplement: Supplementary file 1 — Supplementary Fig. 1 – 6 [file 41746_2024_1018_MOESM1_ESM.docx]

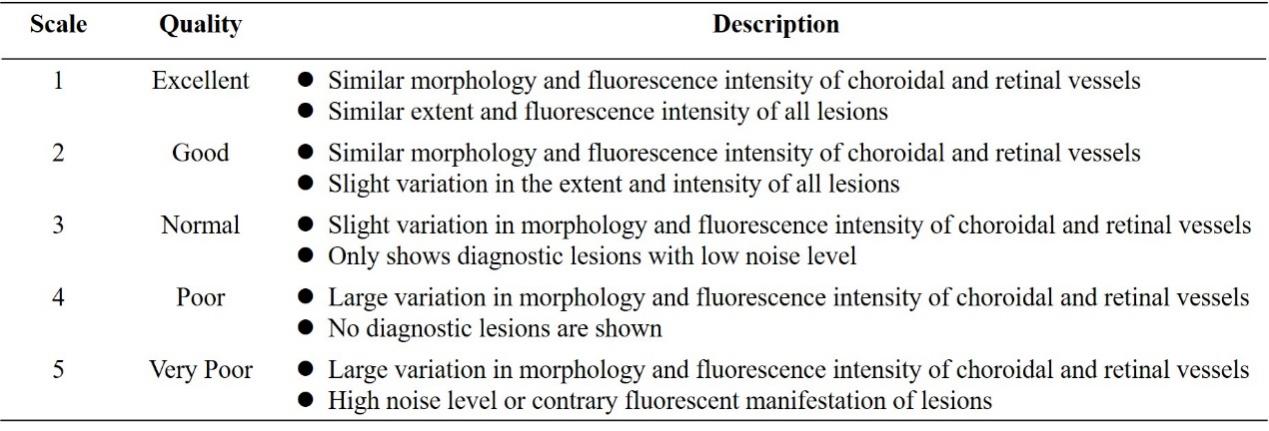


Supplementary Figure 1. Demonstration of the grading criteria. The quality of translated images was evaluated subjectively using a scale of 1 to 5 (1 = excellent, 2 = good, 3 = normal, 4 = poor, and 5 = very poor), with a score of 1 referring to the image quality of the real indocyanine green angiography (ICGA) image. The grading process considered the global similarity, the fidelity of anatomical structures, and the depiction of fluorescence-based pathological lesions.


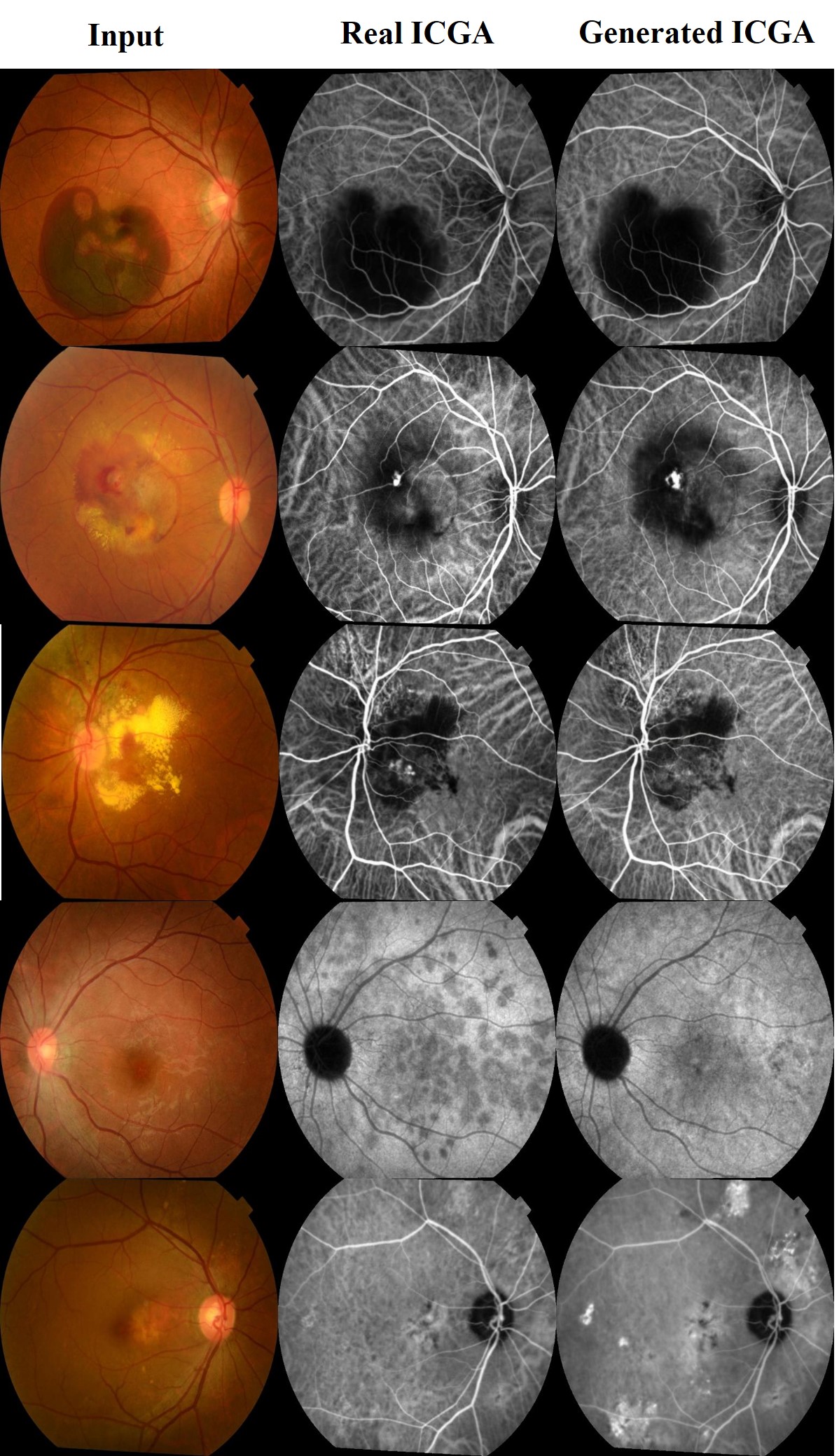


Supplementary Figure 2. Examples of translated indocyanine green angiography (ICGA) images in different qualities. 1^st^ row: excellent, 2^nd^ row: good, 3^rd^ row: normal, 4^th^ row: poor, 5^th^ row: very poor.


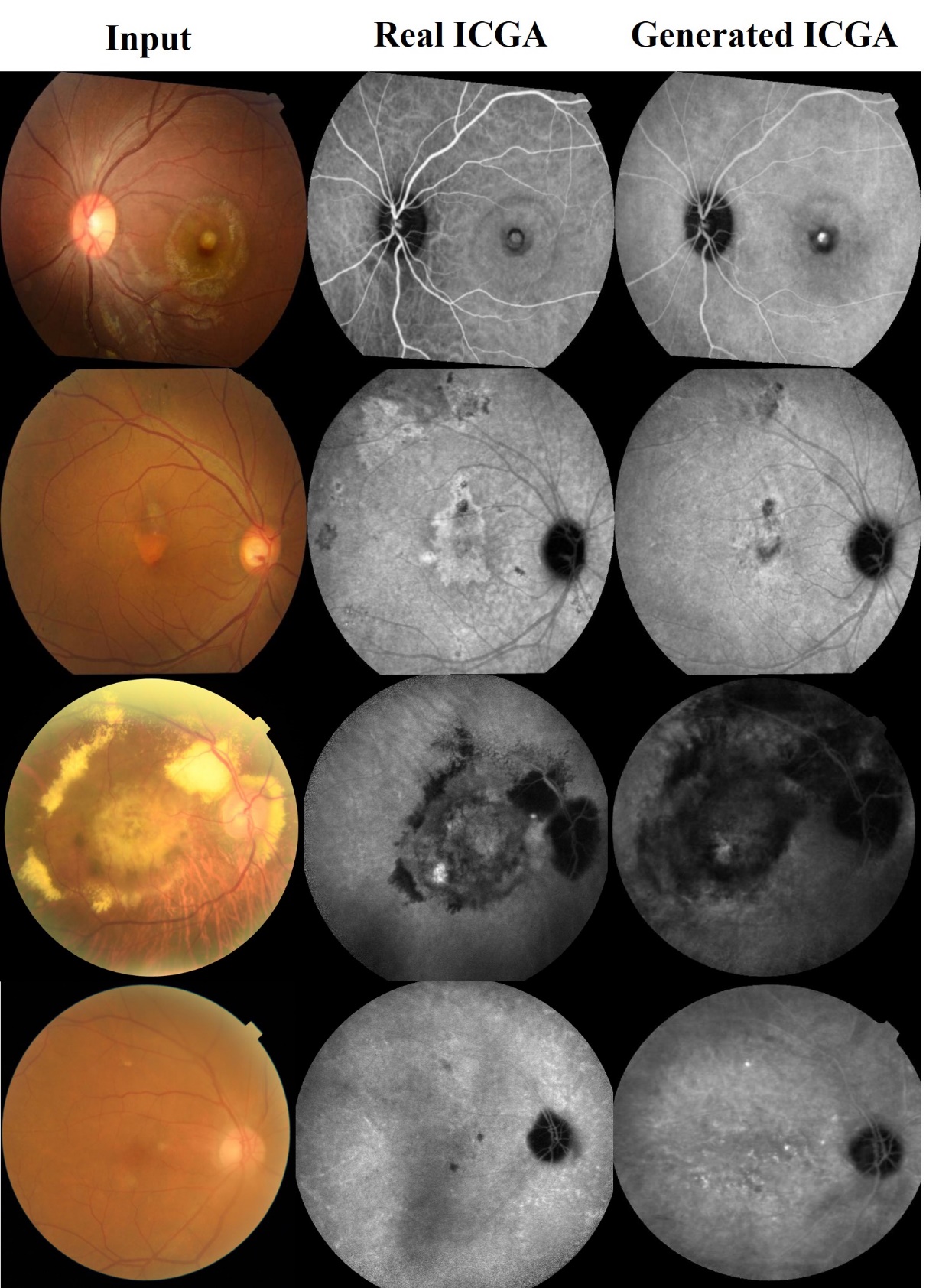


Supplementary Figure 3. Demonstration of unsatisfactory translated indocyanine green angiography (ICGA). 1-2 rows: internal test set. 3-4 rows: external test set. Blurry CF images, thick subretinal hemorrhage and scar may impact the quality of generated ICGA images, presenting as false negative or false positive lesions.


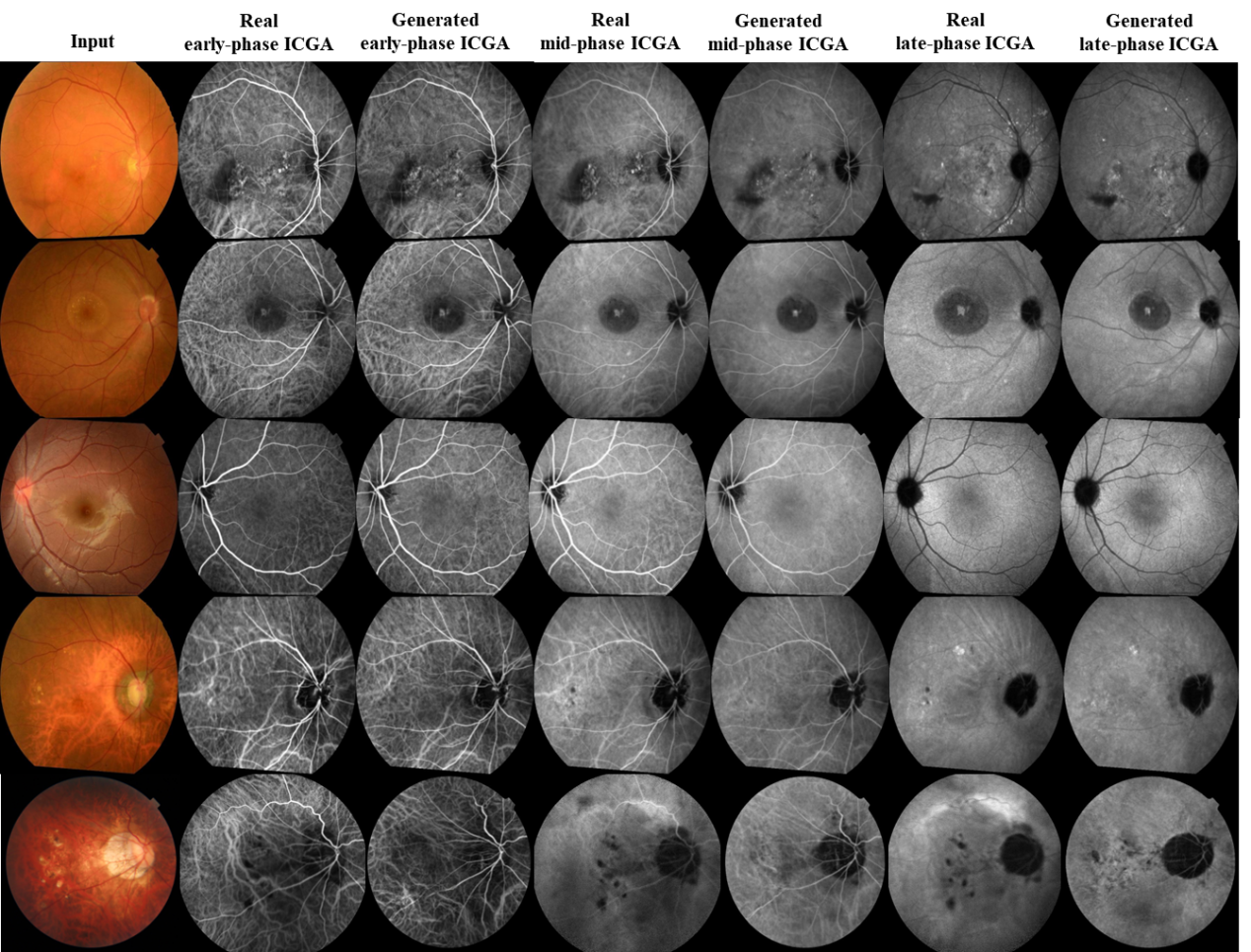


Supplementary Figure 4. Examples of generation for normal fundus and other common chorioretinal diseases. 1^st^ row, polypoidal choroidal vasculopathy, 2^nd^ row, central serous chorioretinopathy, 3^rd^ row, normal fundus, 4^th^ row, pathologic myopia , 5^th^ row, punctate inner choroidopathy.


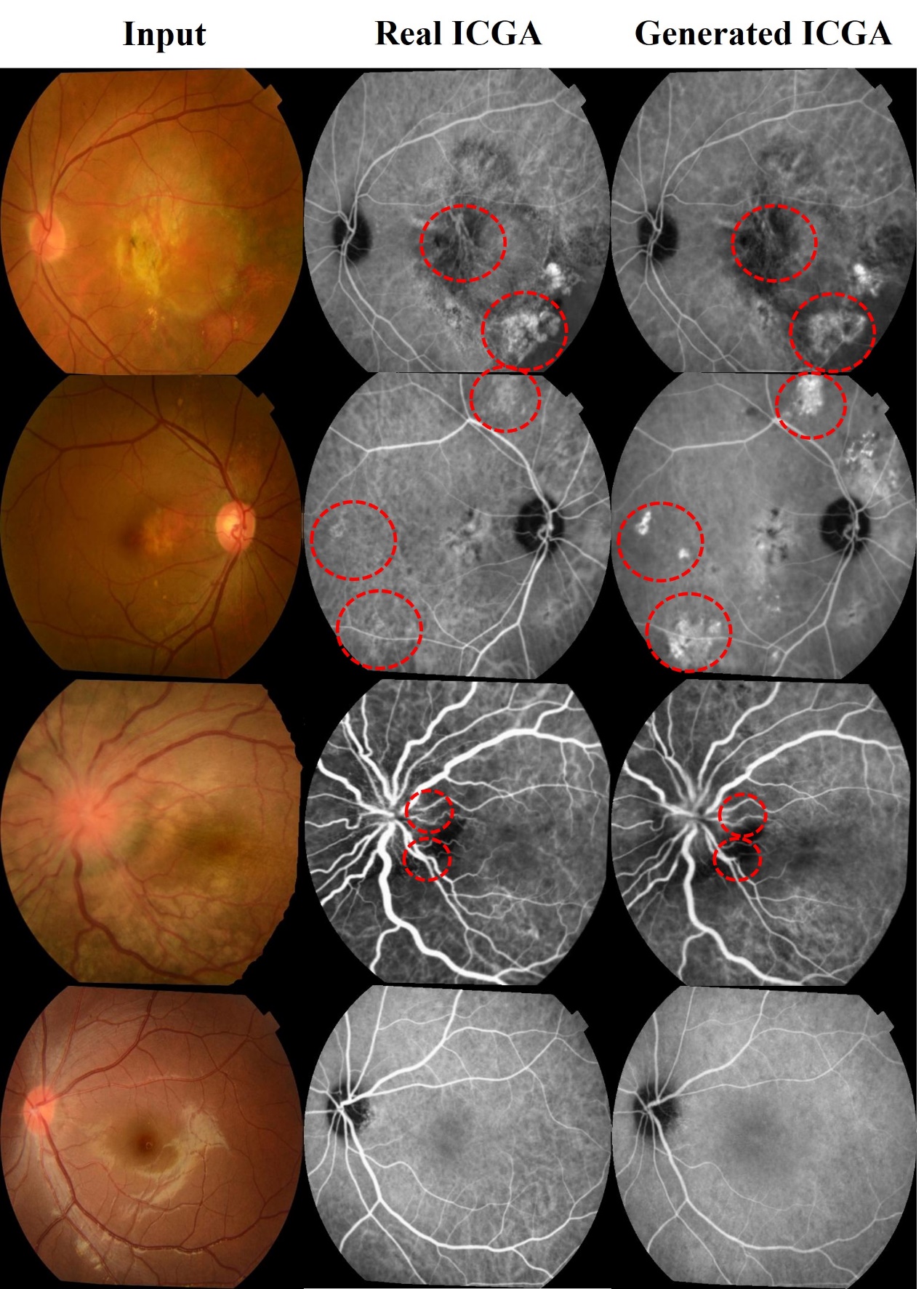


Supplementary Figure 5. Reliable distinguishing features between real and generated images. 1st row, the blurry boundary of lesions, 2nd row, strange lesions that are apparently against established clinical knowledge, 3rd row, vascular discontinuity, 4th row, the blurry texture of choroidal vessels.


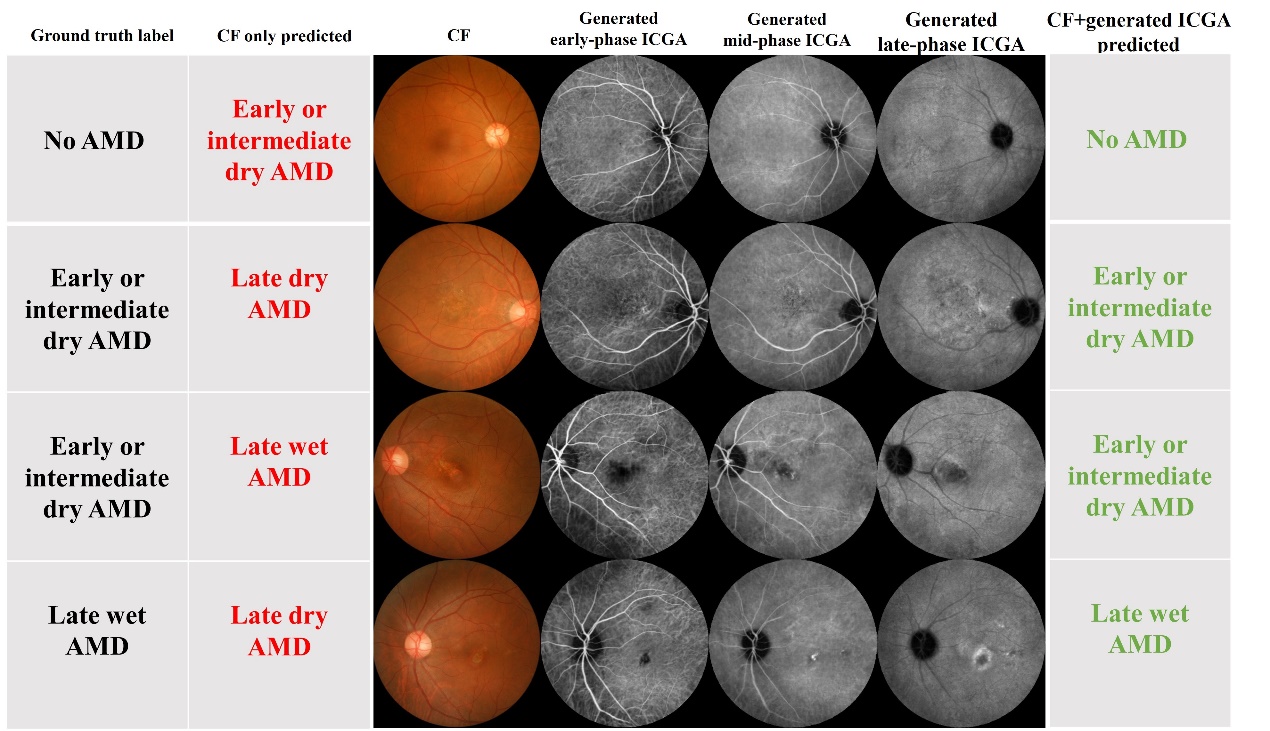


Supplementary Figure 6. Examples of false prediction cases on different age-related macular degeneration (AMD) categories based on color fundus photography (CF) only but were correctly predicted after the addition of translated indocyanine green angiography (ICGA) images. Green annotation: correct prediction. Red annotation: false prediction.
